# Application and research progress of tranexamic acid in the perioperative period of posterior lumbar interbody fusion

**DOI:** 10.3389/fmed.2025.1612281

**Published:** 2025-08-08

**Authors:** Wei Dong, Yuchen Tang, Yu Zhou, Jun Li, Chen Wu, Yin Liu, Yu Yan, Zhenggang Peng, Jun Zhao

**Affiliations:** ^1^Department of Spinal Surgery, Chongqing Orthopedic Hospital of Traditional Chinese Medicine, Chongqing, China; ^2^Department of Orthopedics, The First Affiliated Hospital of Chongqing Medical University, Chongqing, China

**Keywords:** tranexamic acid, posterior lumbar interbody fusion, mode of administration, timing and dose of administration, research progress

## Abstract

Posterior lumbar interbody fusion requires stripping the multifidus muscle, destroying a large amount of cancellous bone and damaging the posterior spinal venous plexus. Typically, surgical trauma is extensive, the surgical duration is long, and the degree of bleeding is substantial. Excessive blood loss can compromise a patient's hemodynamic stability, elevate surgical risks, and cause damage to vital organs, potentially becoming life-threatening in severe cases. Tranexamic acid (TXA) is a lysine derivative that can inhibit fibrinolysis, reduce D-dimer production, and reduce inflammation. In this review, we discuss the application of and research progress on TXA regarding its mechanism of action, mode of administration, timing, dose, safety, and economic benefits. The primary purpose of this review is to provide an essential reference for the administration of TXA during posterior lumbar interbody fusion surgery as well as a reference for future research.

## 1 Introduction

Perioperative blood management refers to the use of multiple techniques for blood protection at various stages of the perioperative period (1). The primary purposes of blood management are to reduce blood loss, blood transfusion rates, and complication rates as well as to improve surgical safety and increase patient satisfaction (2). In the field of spinal surgery, lumbar degenerative diseases account for the majority of diseases requiring posterior lumbar interbody fusion (3, 4). In the past, lumbar degenerative diseases were reported to occur mainly among middle-aged and elderly individuals. In recent years, with the aging of the population and changes in people's lifestyles, the incidence of lumbar degenerative diseases has increased annually, and the age at onset has shown a decreasing trend. Minimally invasive spine surgery can expose the surgical field of vision and can reduce local tissue damage as well as vascular and nerve damage. Furthermore, minimally invasive spine surgery does not require the extensive stripping of muscles, and this technique can retain most of the bone structure and ligament structure (5). According, minimally invasive spine surgery has many advantages. For example, it is beneficial for maintaining the stability of the spine and the nutrition of blood vessels and nerves to the muscles. After minimally invasive spine surgery, postoperative lumbar function recovery is fast. Furthermore, degree of bleeding is low, the low back pain symptoms are mild, and the hospitalization time is short (3, 6, 7). Due to these advantages, minimally invasive spinal surgery is favored by both doctors and patients (8, 9). However, there are many limitations associated with the surgical indications for minimally invasive spine surgery (10). Therefore, posterior lumbar interbody fusion is still the mainstream surgical method for treating lumbar degenerative diseases (9, 11).

The amount of perioperative blood loss among patients requiring posterior lumbar interbody fusion ranges from 670 to 2570 ml (12), and hidden blood loss accounts for ~42%−47% of the total blood loss (12, 13). When no blood loss prevention measures are implemented, the blood transfusion rate ranges from 50 to 81% (12). This large amount of blood loss increases not only the blood transfusion rate but also the infection and complication rates. Moreover, a large amount of blood loss causes changes in patient hemodynamics, thereby affecting the supply of blood to essential organs (14). If substantial blood loss is not treated in time or is improperly controlled, adverse symptoms (such as anemia) may occur, thus affecting the patient's postoperative rehabilitation, quality of life, and prognosis; increasing medical costs; and prolonging hospital stays. In severe cases of blood loss, haemorrhagic shock or even death may occur (15). In addition, blood transfusion may present many risks, such as transfusion reactions, haemolytic reactions, fever, infection, postoperative spinal epidural haematoma formation, acute lung injury, and transfusion-related infectious diseases (16). Furthermore, many previous studies have shown that allogeneic blood transfusion increases the risk of postoperative infection (17), and the lack of blood management resources and high medical expenses have become considerable social burdens. Therefore, optimizing blood management strategies, effectively controlling perioperative bleeding, and reducing blood transfusions have become clinical problems that spinal surgeons urgently need to solve.

To this end, some studies have evaluated the role of patient blood management strategies in intraoperative bleeding and postoperative blood loss during posterior lumbar interbody fusion, including avoiding abdominal compression, controlling hypotension, bipolar coagulation haemostasis, local anesthesia, autologous blood transfusion, haemodilution, intraoperative blood recovery, and the use of iron and erythropoietin (15, 18, 19). These measures are only sometimes effective solutions to the problem of perioperative bleeding in posterior lumbar interbody fusion (20). In addition, tranexamic acid (TXA) is a lysine derivative that can stabilize the structure of fibrin and reduce the incidence of bleeding secondary to hyperfibrinolysis (21). Furthermore, TXA can also reduce the formation of D-dimers (19, 22), and reduce inflammatory responses (23–25). Therefore, TXA is also widely used as a blood management strategy in spinal surgery (21, 26). In this review, the mechanism of action, optimal mode of administration, optimal timing of administration, optimal dose of administration, safety, complications, and economic benefits of TXA during posterior lumbar interbody fusion were discussed.

## 2 Posterior lumbar interbody fusion results in increased blood loss

Posterior lumbar interbody fusion is considered one of the ten significant operations requiring blood transfusion due to the incidence of considerable perioperative blood loss (27). Among these operation, lumbar spinal stenosis and lumbar spondylolisthesis are the most common procedures for posterior lumbar interbody fusion in patients with lumbar degenerative diseases. Posterior lumbar interbody fusion is different from spinal orthopedic surgery. In spinal orthopedic surgery, the primary source of blood loss is only exposed bone surface blood loss. However, in posterior lumbar interbody fusion, the source of blood loss is multifaceted and multifactorial. For example, the complex anatomy of the spine and its rich blood supply, well-fed cancellous bone, and enhanced fibrinolytic activity resulting from surgery may lead to blood loss (28). In addition, patients with lumbar spinal stenosis and/or lumbar spondylolisthesis have a more abundant posterior spinal venous plexus (19). Posterior lumbar interbody fusion makes it difficult to avoid injury to the venous plexus, and the fragile venous plexus cannot contract after damage. An injured venous plexus often leads to difficulty in haemostasis and increased blood loss (29). Moreover, the fibrinolytic response induced by surgical trauma tends to intensify with prolonged operation duration, resulting in increased fibrinolytic hyperfunction. The results of Xie et al. (30) and Blanié et al. (31) indicated that the fibrinolytic effect triggered by surgical trauma peaks at 6 h after surgery and persists for a minimum of 24 h. In conclusion, increased fibrinolytic activity, extensive destruction of cancellous bone, and injury to the posterior spinal venous plexus during posterior lumbar interbody fusion are the primary contributors to perioperative bleeding.

## 3 The mechanism of action of TXA

**Figure 1:**
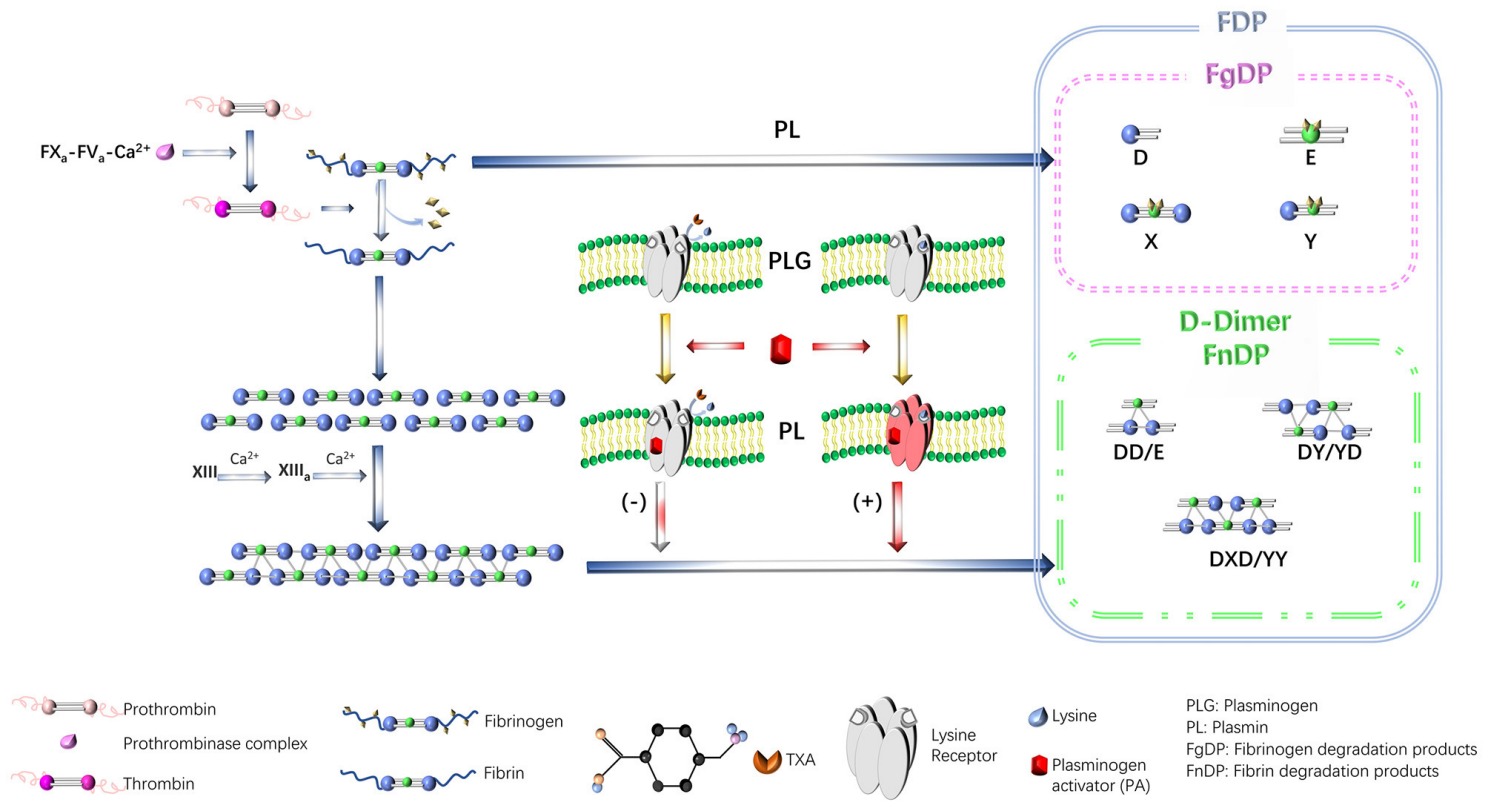
The mechanism of TXA action. TXA, tranexamic acid. Reproduced from ref. (21) with permission from the Wolters Kluwer Health, Inc, copyright 2024.

TXA is a synthetic lysine derivative that was first described in the 1970s. TXA can reversibly and competitively adsorb the lysine binding site (LBS) of the fibrin affinity site on plasmin and plasminogen and form a reversible complex with it. TXA can reduce the conversion of plasminogen to active plasmin, thereby stabilizing the structure of fibrin, reducing the breakdown rate of fibrinogen, and reducing the degree of bleeding secondary to hyperfibrinolysis (21, 32) (Figure 1). In addition, abnormally hyperactive fibrinolytic enzymes inhibit platelet aggregation and decompose coagulation factors; therefore, TXA also has a protective effect on platelets and coagulation factors (19). In addition, TXA can also achieve antiallergic and anti-inflammatory effects by inhibiting vascular permeability, allergic reactions, and the production of kinin as well as other active peptides in inflammatory lesions (23–25). TXA is similar to the antifibrinolytic drug ε-aminocaproic acid (EACA); however, TXA exhibits more efficient and long-lasting antifibrinolytic activity in tissues than EACA. The effect of TXA is 6–10 times stronger than that of EACA and 4–6 times stronger than that of amino-toluic acid (33). In a study by Li et al. (34), the efficacy of TXA was compared with that of aprotinin and EACA among 943 patients who underwent spinal surgery. The findings revealed that all antifibrinolytic drugs can reduce perioperative blood loss and reduce the need for blood transfusions. However, TXA was more effective than aprotinin or EACA. In addition, Li et al. (34) also reported that TXA can reduce intraoperative blood loss by 53%, reduce postoperative blood loss by 20%, and reduce transfusion volume by 62%.

TXA is metabolized by the kidney and has a half-life of ~80–120 min, reaching a peak plasma concentration after 60 min of intravenous infusion. Approximately 30%−55% of TXA is excreted through the kidney 1–3 h after administration, and ~76%−90% of TXA is excreted through the kidneys in its original form through the urine within 24 h of administration (35). Therefore, for patients with renal insufficiency, the dose of TXA must be appropriately adjusted. Moreover, an in vitro study revealed that the minimum effective plasma concentration of TXA was 5–10 mg/L, and a plasma concentration of 10 mg/L could inhibited fibrinolysis by 80% (35). This critical finding provides a fundamental theoretical basis for future studies of the optimal timing and dosage of TXA.

## 4 The clinical application of TXA

TXA, a haemostatic agent, has gained widespread approval and clinical use after the ongoing controversy related to aprotinin, which was initially discontinued in 2007 (36). TXA was also approved by the U.S. Food and Drug Administration (FDA) and included in the World Health Organization's list of essential drugs in 2011 (36). TXA has been shown to reduce bleeding in a variety of surgeries, including heart surgeries, trauma surgeries, joint replacement surgeries, craniocerebral injury surgeries, and gynecological and urological surgeries (37–45). Moreover, TXA has also been used in nonsurgical applications, such as for the treatment of leukemia-related bleeding, eye-related bleeding, nose-related bleeding, repeated haemoptysis, gastrointestinal bleeding, menorrhagia, melasma, and other problems (46–49).

### 4.1 The mode of TXA administration

The application of TXA in total knee arthroplasty has been very mature, including intravenous administration (41), local administration (42), oral administration (40, 43, 44), and combination therapy (45). However, compared with total knee arthroplasty, posterior lumbar interbody fusion often requires the exposure of the spinal cord. When the spinal cord is immersed in TXA, the concentration of TXA in the cerebrospinal fluid increases, which can lead to epilepsy or neurotoxicity (36, 50, 51). Therefore, intravenous administration of TXA is preferred for posterior lumbar interbody fusion.

Some previous studies have shown that (52), local or intravenous administration of TXA can reduce perioperative blood loss. Furthermore, TXA does not increase the risk of thrombosis. Moreover, some researchers believe that local administration of TXA can provide the maximum drug concentration at the bleeding site, thereby minimizing systemic absorption. However, the fibrinolytic system caused by surgical trauma is usually activated at the beginning of surgery, and it releases a large amount of fibrinolytic enzyme to decompose fibrin. Compared with local administration after the activation of the fibrinolytic system, intravenous injection of TXA before the beginning of surgery can stabilize the structure of fibrin by inhibiting the activation of the fibrinolytic system in advance, improving the speed of haemostasis and enhaninge haemostasis (53, 54). Therefore, TXA should be intravenously administered before the start of posterior lumbar interbody fusion.

### 4.2 Timing and dosage of TXA administration

TXA is considered to be effective in terms of reducing perioperative blood loss. However, it remains unclear whether TXA is routinely used in posterior lumbar interbody fusion. Furthermore, the optimal timing and dosage of TXA administration remain unclear. The fibrinolytic system is activated by surgical trauma. Fibrinolytic activation is a cascade reaction process. Therefore, choosing the appropriate timing for TXA administration is important. Most scholars believe that TXA should be used before fibrinolytic activation (41, 53).

Related studies have shown that the half-life of TXA is ~2 h, and the effective plasma concentration is 1 μg/ml. After an intravenous infusion of 15 mg/kg, TXA exerts the strongest effects within the first 16 h after administration (55). In addition, Abdou et al. (15) reported that a single intravenous infusion of TXA (10 mg/kg) did not significantly reduce the amount of blood loss or blood transfusion during spinal fusion surgery. However, Tsutsumimoto et al. (55) found that intravenous infusion of TXA (15 mg/kg) before skin incision significantly reduced the amount of bleeding in the first 16 h after surgery (~37%).

To better determine the optimal dose of TXA, Brown et al. (56) analyzed 11 articles related to the clinical application of TXA in spinal fusion surgery. Data from 411 patients revealed that the most common route of administration was intravenous administration, the most common timing of administration was preoperative administration, and the most common dosage was 15 mg/kg. By data from 2,042 patients undergoing total knee arthroplasty, Wilde et al. (57) concluded that intravenous infusion of 1 g TXA has the same haemostatic effect as a dose of 2 g, indicating that higher doses have no additional benefits. Although the study by Wilde et al. was focused on total knee arthroplasty, the basic mechanism of action of TXA is similar in different surgeries, which is to reduce bleeding by inhibiting the fibrinolytic system. Moreover, some studies related to spinal surgery have shown that intravenous administration of 1 g of TXA also has a certain effect in reducing bleeding during spinal surgery, which is somewhat similar to its effect in total knee arthroplasty (58). Therefore, it is reasonable to refer to this dosage in spinal surgery to a certain extent. Although TXA has a similar mechanism of action in reducing surgical bleeding, the optimal dosage used in total knee arthroplasty cannot be directly applied to lumbar surgery due to potential differences in the surgical anatomy between posterior lumbar interbody fusion and total knee arthroplasty. The feasibility of this approach requires further validation. In addition, Lin and Xiaoyi (59) analyzed data from 26,079 patients who used TXA and 7,395 patients who did not use TXA. The found that the prevalence of TXA induced epilepsy was 2.7%, and this prevalence increased with increasing TXA dose. Thus, low-dose TXA (10–15 mg/kg or 1 g) is considered a relatively safe and effective dose in posterior lumbar interbody fusion. Compared with high-dose TXA, it has a lower risk of causing epilepsy. However, due to factors such as individual differences, the possibility of low-dose TXA triggering epilepsy still cannot be completely ruled out. Clinicians still need to closely monitor the patient's condition when using it. Some studies have shown that high-dose TXA (10–100 mg/kg) is superior to low-dose TXA (< 10 mg/kg) in significantly reducing intraoperative blood loss (60). Currently, the commonly accepted intravenous dose of TXA is 10–15 mg/kg or 1 g. This is mainly because this dose is relatively common in clinical applications and is relatively safe. It can not only reduce bleeding to a certain extent but also control the risk of complications such as epilepsy (61). Nevertheless, there is still a lack of sufficient evidence regarding the optimal and safe dosage, and more high-quality studies are needed for further determination. In addition, hyperfibrinolysis caused by surgical trauma often lasts for at least 24 h after surgery (30, 31). Moreover, hidden blood loss accounts for a large proportion of postoperative blood loss (12, 13). Although a single preoperative dose or a single preoperative dose combined with an intraoperative maintenance dose can reduce bleeding to a certain extent, they may not completely inhibit the entire hyperfibrinolysis process. Therefore, it is necessary to further study the effect of continuous postoperative use of different doses of TXA on reducing blood loss in the future, in order to determine a more effective dosing regimen.

## 5 Safety and complications of TXA

The application of TXA during spinal surgery has been extensively studied. However, up to now, TXA is not routinely used in China. Because of the lack of high-quality, large-sample, multicenter clinical research articles and the lack of statistical analyses of previously reported studies, there are safety concerns with respect to TXA. These safety concerns include an increased incidence of thromboembolic events [such as pulmonary embolism (PE), deep vein thrombosis (DVT), and myocardial infarction (MI)] as well as increased incidence of epilepsy that can occur due to a single moderate or high dose of TXA (15, 61).

To confirm the safety of TXA, Yuan et al. (62) conducted a meta-analysis of 685 patients who underwent scoliosis surgery; none of these patients experienced thromboembolic events. Brown et al. (56) analyzed date from 411 patients who underwent spinal fusion surgery and concluded that TXA significantly reduced perioperative blood loss and did not increase the risk of related complications. Furthermore, in a meta-analysis by Neilipovitz et al. (63), only 1 case of deep vein thrombosis was observed in the control group. A meta-analysis by Chornenki et al. (64) revealed that there was no increased risk of venous or arterial thrombosis in nonsurgical patients receiving systemic TXA treatment (illustrated in Table 1). These studies indicated that intravenous application of TXA in spinal surgery is safe and does not increase the risk of thromboembolic events. The safety of TXA may be related to the fact that the inhibitory effect of TXA on fibrinolysis is mainly limited to surgical wounds rather than the circulatory system; therefore, TXA does not affect the venous wall (65). In addition, TXA does not increase the formation of epidural blood clots, which would increase the risk of nerve injury (28).

**Table 1:** Systematic review of 22 RCTs (including CRASH-2 and WOMAN).

**Complication**	**Number of trials (*n*)**	**Weighted event rates**		**RR (95% CI)**	***I* ^2^**
		**TXA**	**No TXA**		
DVT	8 (46,630)	0.28%	0.29%	0.97 (0.69–1.37)	0%
PE	6 (43,161)	0.52%	0.54%	0.97 (0.75–1.26)	0%
MI	3 (42,470)	0.27%	0.30%	0.88 (0.43–1.84)	46%
Stroke	5 (42,815)	0.45%	0.41%	1.10 (0.68–1.78)	31%

DVT, Deep vein thrombosis; PE, Pulmonary embolism; MI, Myocardial infarction.

However, when it is administered via intravenous injection, TXA can penetrate the blood-brain barrier and affect the central nervous system (CNS) and the eyes. The concentration of TXA in cerebrospinal fluid and aqueous humor is ~10% of its concentration in plasma (51). During cardiopulmonary bypass (CPB) surgery, administering a large dose of TXA (≥100 mg/kg) intravenously can lead to epilepsy and postoperative convulsions in some susceptible patients (66). Similar experimental studies have shown that when TXA is administered locally in central nervous system tissues, it may exert neurotoxic effects by interfering with central GABA-A and glycine receptors (36, 51). According to relevant studies, TXA also has some rare but severe side effects, such as visual impairment and acute renal cortical necrosis (67).

**Figure 2:**
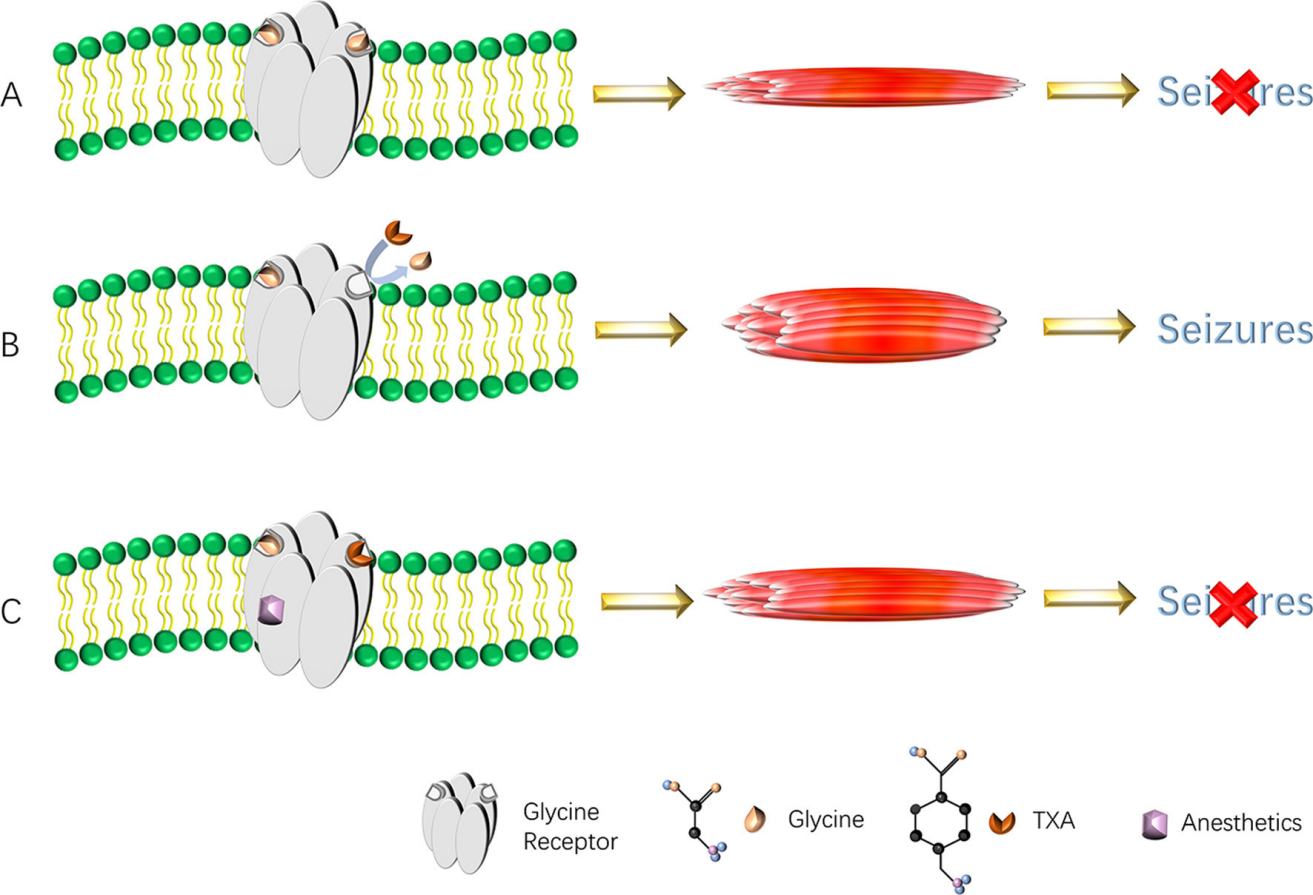
**(A–C)** Diagram of TXA causing seizures. TXA binds to the glycine receptors, resulting in a decrease in inhibitory current. This reduction in anion conduction increases excitability, which gives rise to seizures. Anesthetics reverse the effect of TXA by increasing glycine receptor function and thereby prevent or reverse TXA-induced seizures. TXA, tranexamic acid.

Larger doses of TXA can also cause non-ischemic seizures. When glycine (Gly) binds to the Gly receptor, it does not induce seizures (Figure 2A). TXA competitively binds to glycine receptors and inhibits their activation, thereby increasing muscle excitability and leading to non-ischemic seizures (36) (Figure 2B). When the concentration of TXA is low, glycine and GABA-A receptor agonists compete for the binding site of the glycine receptor, which does not cause seizures. In addition, anesthetics (such as propofol, sevoflurane, and desflurane) are positive allosteric modulators of the glycine receptor, which can enhance the function of the glycine receptor and reverse the inhibitory effect of TXA after binding to the glycine receptor, thereby preventing seizures (68) (Figure 2C). Typically, 5–8 h after surgery, the level of anesthesia in the central nervous system drops rapidly, while the level of TXA either remains at its peak or gradually decreases slowly. Therefore, seizures often occur during this period. Based on the above mechanism, we speculate that the use of TXA in posterior lumbar interbody fusion may lead to the possibility of epilepsy, and spinal surgeon must be aware of risk. When epilepsy occurs, the inhibitory effect of TXA binding to glycine receptors can be reversed by intravenous injection of propofol or midazolam. As there are currently few detailed clinical studies on the association between TXA use and epileptic seizures, future research is expected to focus on the risk stratification of TXA-induced epilepsy. This will involve identifying high-risk individuals (such as patients with renal insufficiency, pre-existing epilepsy, or those undergoing specific neurosurgical procedures) and suggest actionable monitoring protocols to mitigate this adverse effect.

## 6 The economic benefits of TXA

To better clarify the economic benefits of TXA, a previous study conducted a statistical analysis of patients who underwent total hip arthroplasty. TXA was found to result to savings of at least $128 per patient and up to ~$600 among patients undergoing revision surgery (69). Similarly, Ehresman et al. (70) analyzed data from 1,353 patients who underwent lumbar fusion surgery and reported that TXA led to savings of ~$328.69 per patient and resulted in fewer allogeneic blood transfusions during hospitalization. Due to the considerable blood loss that occurs after posterior lumbar interbody fusion, patients often need blood products (12), which increases the economic burden of the surgery. Therefore, if TXA is used, it is expected to reduce patients' hospitalization expenses and alleviate the shortage of blood resources.

## 7 Conclusion and future prospects

During the perioperative period of posterior lumbar interbody fusion, doctors usually need to consider blood conservation strategies for patients. Intravenous use of TXA during perioperative period can reduce blood loss in patients without increasing the incidence of thromboembolic events (15, 53, 54). Therefore, the administration of TXA during posterior lumbar interbody fusion is very promising. However, to date, the administration details of TXA in posterior lumbar interbody fusion remains controversial. These controversies mainly revolve around the use, timing, and dose of TXA. In this manuscript, the application of TXA during spinal surgery was deeply explored, and new insights were put forward regarding the existing controversial issues (illustrated in Table 2). The findings were as follows. (1) The haemostatic effect of preoperative, intravenous administration of TXA was better than that of local administration. (2) The first dose of TXA should be administered before the activation of the fibrinolytic system. (3) The recommended dose of TXA is 10 to 15 mg/kg or 1 g; higher doses have no additional effects on increasing haemostasis. (4) The hyperfibrinolysis process caused by surgical trauma lasts for at least 24 h after the operation. The directions for further improvement of current research are as follows: (1) Clinical research validation: More high-quality, multicenter, and large-sample prospective clinical studies are needed to confirm the reliability of existing research findings. (2) Optimization of administration protocols: A randomized controlled clinical trial design combined with a long-term efficacy follow-up system was used to systematically explore the optimized administration regimen of TXA in posterior lumbar interbody fusion. This specifically covered the precise control of medication timing (such as different time points before, during, or after surgery), the scientific setting of dose gradients (including the reasonable ratio of loading dose to maintenance dose), and the comparative analysis of administration routes (intravenous infusion, local application, or combined administration). (3) Establishment of predictive models: Construct clinical predictive models for such surgeries to identify key influencing factors of TXA in reducing perioperative blood loss. (4) Expansion of application scope: Conduct in-depth studies on the efficacy of TXA in various spinal surgeries (such as cervical and thoracic surgeries) to verify its universality. (5) Analysis of risk factors: Analyze the risk factors of perioperative blood loss after posterior lumbar interbody fusion to achieve individualized and rational use of TXA.

**Table 2:** Major studies on the mode of administration, timing, and dosage of TXA.

**Study**	**Research object**	**Groups**	**Conclusion**
Hui et al., 2021 (52)	Cervical, thoracic, and lumbar spinal surgery patients	Topical use of TXA vs. Control group	Topical use of TXA in spinal surgery can reduce blood loss
Mu et al., 2018 (53)	Double-segment posterior lumbar interbody fusion (PLIF)	Intravenous administration group vs. topical administration group vs. placebo group	TXA can reduce blood loss, extubation time, and the length of hospital stay. In addition, intravenous administration can minimize blood loss more efficiently
Cao et al., 2022 (54)	Patients undergoing spinal surgery	Intravenous plus topical administration of TXA vs. topical administration vs. placebo group	Effect of reducing blood loss: intravenous plus topical administration > topical administration > Placebo group
Abdou et al., 2022 (15)	interbody fusion (PLIF)	Intravenous infusion of 10 mg/kg for 20 min after induction of anesthesia + the maintenance dose of 1 mg/kg/h vs. no receive TXA	Low-dose TXA has no effect on the reduction of intraoperative blood loss volume or blood transfusion requirements. However, it can significantly reduce the need for postoperative blood transfusion requirements
Tsutsumimoto et al., 2011 (55)	“French-door” cervical laminoplasty from C3 to C6 was performed	Patients received 15 mg/kg body weight of TXA before the skin incision was made vs. placebo group	Intravenous infusion of TXA can significantly reduce the amount of bleeding in the first 16 h after surgery (~37% reduction)
Hui et al., 2018 (60)	Spinal surgeries: A meta-analysis	High-dose TXA (10–100 mg/kg) vs. low-dose TXA (<10 mg/kg)	High-dose TXA significantly reduces intraoperative-perioperative allogeneic transfusion rates and operative time
Brown et al., 2022 (56)	Cervical, thoracic, and lumbar laminectomy and fusion	A total of 411 patients from 11 relevant clinical studies were analyzed	The most common route of administration, timing, and dose of TXA was preoperative intravenous injection at a dose of 15 mg/kg
Lin et al., 2016 (59)	TXA associated seizures	26,079 patients with TXA exposure vs. 7,395 patients without TXA exposure	The prevalence of TXA-induced epilepsy was 2.7%, and this rate increased as the TXA dose increased
Cheriyan et al., 2015 (61)	Efficacy of TXA on surgical bleeding in spine surgery	Intravenous TXA vs. placebo group	Intravenous injection of TXA at a dose of 10–15 mg/kg or 1 g is sufficient, and a higher dose does not yield additional haemostatic benefits but does increase the risk of epilepsy
